# Evaluation of the ‘Spaarne soft tissue procedure’ as a treatment for recurrent patellar dislocations: a four-in-one technique

**DOI:** 10.1186/s40634-021-00349-8

**Published:** 2021-04-20

**Authors:** Raymond Puijk, Rachid Rassir, Jan K. G. Louwerens, Inger N. Sierevelt, Tjitte de Jong, Peter A. Nolte

**Affiliations:** 1 Department, of Orthopaedic Surgery, Spaarne Gasthuis, Spaarnepoort 1, 2134 TM, Hoofddorp, the Netherlands; 2 Specialized Center of Orthopedic Research and Education (SCORE), Xpert Orthopedics, Amsterdam, Laarderhoogtweg 12 The Netherlands

**Keywords:** Recurrent patellar dislocations, Patellar dislocation, Patellar instability, Treatment, Soft tissue procedure, Spaarne soft tissue procedure, Patella, Redislocation, Adolescents, Adults

## Abstract

**Purpose:**

The ‘Spaarne soft tissue procedure’, is a 4-in-1 soft tissue procedure that treats recurrent patellar dislocations in the young and active population. The procedure has not yet described elsewhere. The purpose of this study is to analyse the redislocation rate and to evaluate the postoperative knee function and patient satisfaction.

**Methods:**

Twenty-seven patients (34 knees) underwent the four-in-one SST-procedure. The 4-step technique required a minor change in 2010, including the use of a smaller strip of the patellar tendon for transposition. After a median follow-up of 10.4 years, the redislocation rate was evaluated as the primary outcome measure. Secondary outcome measures were functional outcome (IKDC, Kujala, Lysholm and Tegner activity scale) and Numeric Rating Scales for satisfaction and pain.

**Results:**

Redislocation occurred in 8 cases (23.5%) and subluxation occurred in 13 cases (38.2%) post-surgery. A significant higher number of redislocations and subluxations were seen before 2010 (*p* = 0.04, *p* = 0.03). The median postoperative IKDC, Lysholm and Kujala scores for the total group were 54, 76 and 81 respectively. Pre- and postoperative Tegner activity scale were both level 3. Median NRS scores during rest, walking and sports were 1, 3 and 5 respectively. Satisfaction with the procedure was reported as ‘excellent’ or ‘good’ by 79% of the patients.

**Conclusion:**

Despite the high overall redislocation rate and increased pain scores, the SST-procedure shows to be a safe procedure in patients with recurrent patellar dislocations based on the cases after 2010. Mid- and long-term results show moderate to good functional outcomes and satisfaction.

**Level of evidence:**

Therapeutic retrospective cohort study, LEVEL III

## Introduction

Primary patellar dislocation has an average annual incidence of 5.8–7 cases per 100.000, most common in the young and active population [22]. In more than 70% of cases, it occurs during sports activities and almost always results in a lateral dislocation of the patella [16, 21, 22]. After a first-time patellar dislocation, the redislocation rate varies between 15 and 80%, which increases to more than 50% after a second dislocation [16, 21, 22]. Furthermore, subluxations of the patella are also more common after a primary dislocation. A sustained patellar dislocation can result in pain, instability, recurrent patellar dislocations (RPD), decreased activity, articular cartilage lesions and eventually patellofemoral arthritis [16, 22].

When recurrent patellar dislocations or subluxations occur, patellofemoral instability is likely to be present. This instability may be due to damage to the anatomical stabilizing structures because of an earlier traumatic patellar luxation, or a predisposing vulnerability in the patellofemoral joint, like soft tissue pathology, patellar alta or trochlear dysplasia [8]. Primary patellar dislocation is most often treated conservatively with a short duration of immobilization by means of a brace/tape, followed by strengthening of the quadriceps muscles and range of motion training [3, 22]. In contrast, most recurrent patellar dislocations are treated surgically as they are frequently based on a malaligned limb, osseous deformities or soft-tissue damage [22]. Surgical patellar stabilizing procedures can be divided into two groups: osseous realignment procedures and soft tissue procedures [16, 22]. The medial patellofemoral ligament (MPFL) reconstruction is the most widely described soft tissue procedure, along with the Roux-Goldthwait procedure and the three-in-one technique. They all serve the purpose of stabilizing the patellofemoral joint by inducing a medializing force [12–15, 17, 22]. The SST procedure modifies and combines these techniques without using a semitendinosus/gracilis or allograft. This makes the procedure less invasive, and also fit for the paediatric population due to the lack of any osseous corrections.

The purpose of this study is to describe the operative technique and clinical outcome of a novel four-in-one technique: the Spaarne Soft Tissue Procedure (SST). It was first performed in 2001 at a tertiary hospital in the Netherlands to treat patients with recurrent patellar dislocations. The procedure consists of 4 steps: 1) releasing the lateral retinaculum, 2) reinforcement of the MPFL by utilizing a strip of the medial retinaculum, 3) transferring the lateral third (before 2010) or quarter (after 2010) of the patellar tendon (PT) to the medial side, 4) reefing the gab of the medial retinaculum.

The primary objective of the current study is to analyse the redislocation and subluxation rate of this procedure. The secondary objective is to evaluate post-operative knee function and patient satisfaction.

## Material and methods

### Study design

The study was designed as a retrospective cohort study. All patients provided written informed consent and ethical approval by the Internal Review Board (ACLU# 2019.0092) was obtained.

### Study population

All patients who were treated with the Spaarne Soft Tissue (SST) procedure between January 2001 and January 2019 were asked to participate in the study. The procedures were performed by two surgeons (PN, TdJ), all patients were treated in the same tertiary-care hospital. Patients after January 2010 underwent the same surgical technique but with a small adjustment based on the surgeon’s judgment. Inclusion criteria were patella instability with recurrent patellar dislocations not responding to full conservative treatment. Skeletally mature as well as skeletally immature patients were included. Patients with clinically relevant skeletal deformities (including patella alta, trochlear dysplasia or cartilage defects) or previous surgical patellar re-alignment procedures were excluded. However, patients with clinically irrelevant mild skeletal deformity, were accepted for the operation to the opinion of the operating surgeon. The presence of skeletal deformities was assessed by means of physical examination (Q-angle, apprehension test and J-sign) and radiographic assessment (posteroanterior, lateral and an axial patellofemoral radiograph). If there was any doubt about the presence of a clinically relevant skeletal abnormality, axial imaging by CT or MRI was performed to provide to diagnose and/or classify this abnormality.

### Data collection

All patients were contacted by telephone by the first author (RP) and asked if they were willing to participate. Data was collected through medical records and validated questionnaires. After providing their informed consent, patients were asked to complete a number of questionnaires. This included the International Knee Documentation Committee Subjective Knee evaluation (IKDC) [6], Lysholm [4, 19], Kujala [7, 20], SF-36 [1], and two Tegner questionnaires (preoperative and postoperative) [4, 19]. The IKDC, Lysholm and Kujula are focused on functional impairment and subjective symptoms and have a score range from 0 (worst outcome) to 100 (best outcome). The SF-36 is aimed at overall health related to quality of life and consists of a scale of 0 (worst) to 100 (best outcome). The Tegner questionnaires evaluates the level of work- and sports-activity on a 0 (worst) to 10 (best) level scale. The number of recurring dislocations and subluxations as well as additional symptoms like swelling, instability and crepitus were also registered. Pain was evaluated with a Numerical Rating Scale (NRS, 0 = no pain and 10 = severe pain) and the patient’s self-reported change in pain and daily functioning were evaluated with a 6-point Likert scale (much worse, slightly worse, not changed, slightly improved, improved, much improved). Finally, overall satisfaction was evaluated with a 4-point likert scale (bad, reasonable, good, excellent).

### Operative technique

**Fig. 1:**
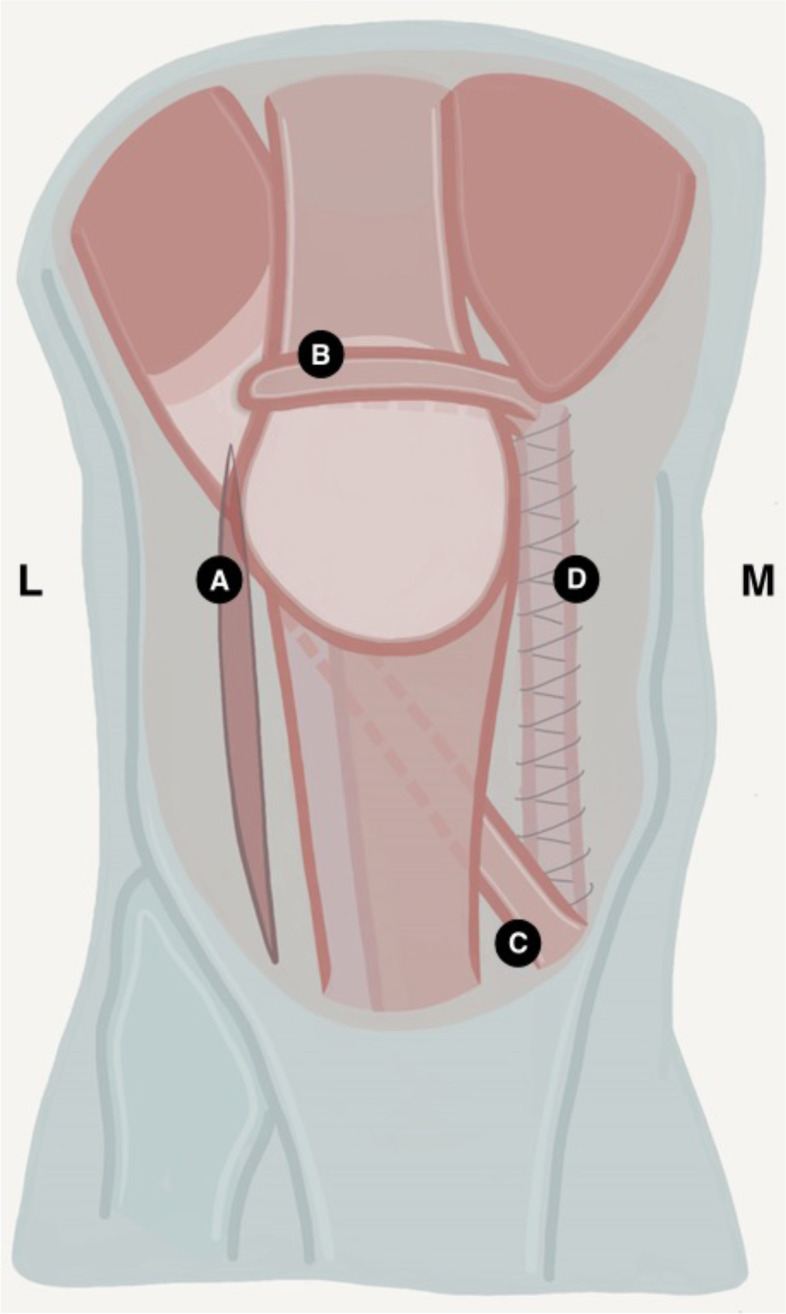
Schematic illustration of the 4 steps of the SST procedure after 2010. The four steps of the SST-Procedure: **a** Releasing the lateral retinaculum. **b** Releasing a strip of the medial retinaculum and transferring the distal part as a loop around the quadriceps tendon after which it is attached to the proximal part. **c** Releasing the distal lateral quarter of the patella tendon and transferring it below the patella tendon to attach it to the medial side of the tuberositas tibiae. **d** Reefing the medial retinaculum

Surgery was performed under general or spinal anaesthesia. Patients were operated in the supine position and a tourniquet was used. An 8–10 cm median skin incision was performed, starting just proximal of the patella and ending just below the insertion of the PT. The subcutaneous tissue was mobilized, taking care of infrapatellar nerve branches. Adequate exposure was achieved once the peritendineum overlying the PT and the medial and lateral retinacula was clearly visualized. The procedure consists of four steps and is drawn schematically in Fig. 1. The procedure starts with a lateral retinacular release over the course of several centimetres. The release was adequate if it was possible to shift the patella medially. In the next step, a 5-to-8-mm strip of the medial retinaculum was released, leaving the proximal attachment intact. The strip was tunnelled under the quadriceps tendon, brought into a loop back over the tendon and got attached to the proximal part of the strip with braided absorbable sutures (Vicryl, Ethicon Inc, Somerville, NJ, USA). The strip as a loop serves as a reinforcement of the MPFL. Subsequently, the peritendineum of the PT was opened and the distal lateral third (before 2010) or quarter (after January 2010) of the PT was vertically incised along its entire length from the apex of the patella to the insertion at the tuberositas tibiae. The proximal origin of this strip was left intact, but the distal insertion at the Tuberositas tibiae was detached and passed under the medial part and attached to the medial side of the tibia. Finally, the cut of the medial retinaculum was approximated by relatively tightening the medial side equal to reefing with the earlier used absorbable Vycril suture.

Postoperatively, patients received a knee immobilizing splint for 6 weeks, and were allowed full weight-bearing. Rehabilitation consisted of muscle-setting and active flexion up to 90° of flexion for 6 weeks under direct supervision of a physical therapist. After 6 weeks, patients were allowed to progress more strength and train function. All patients over the age of 16 were given a low molecular weight heparin before surgery as thrombosis prophylaxis.

### Statistical analysis

All collected data was entered into a database. (Research Manager, Cloud9 software, Deventer, Netherlands). Due to the small sample size, all collected data was considered non-normally distributed and was therefore consistently presented as medians with accompanying interquartile ranges. Sub-analysis was performed by use of Mann–Whitney U tests and Chi^2^-tests (or Fisher Exact test when appropriate) to assess the association of redislocation and subluxations with functional and subjective outcomes. Statistical significance was considered when *P* value were 0.05 at a 95% confidence of interval. Statistical analysis was performed using SPSS statistics software (IBM, Armonk, NY, USA, version 26.0).

## Results

**Fig. 2:**
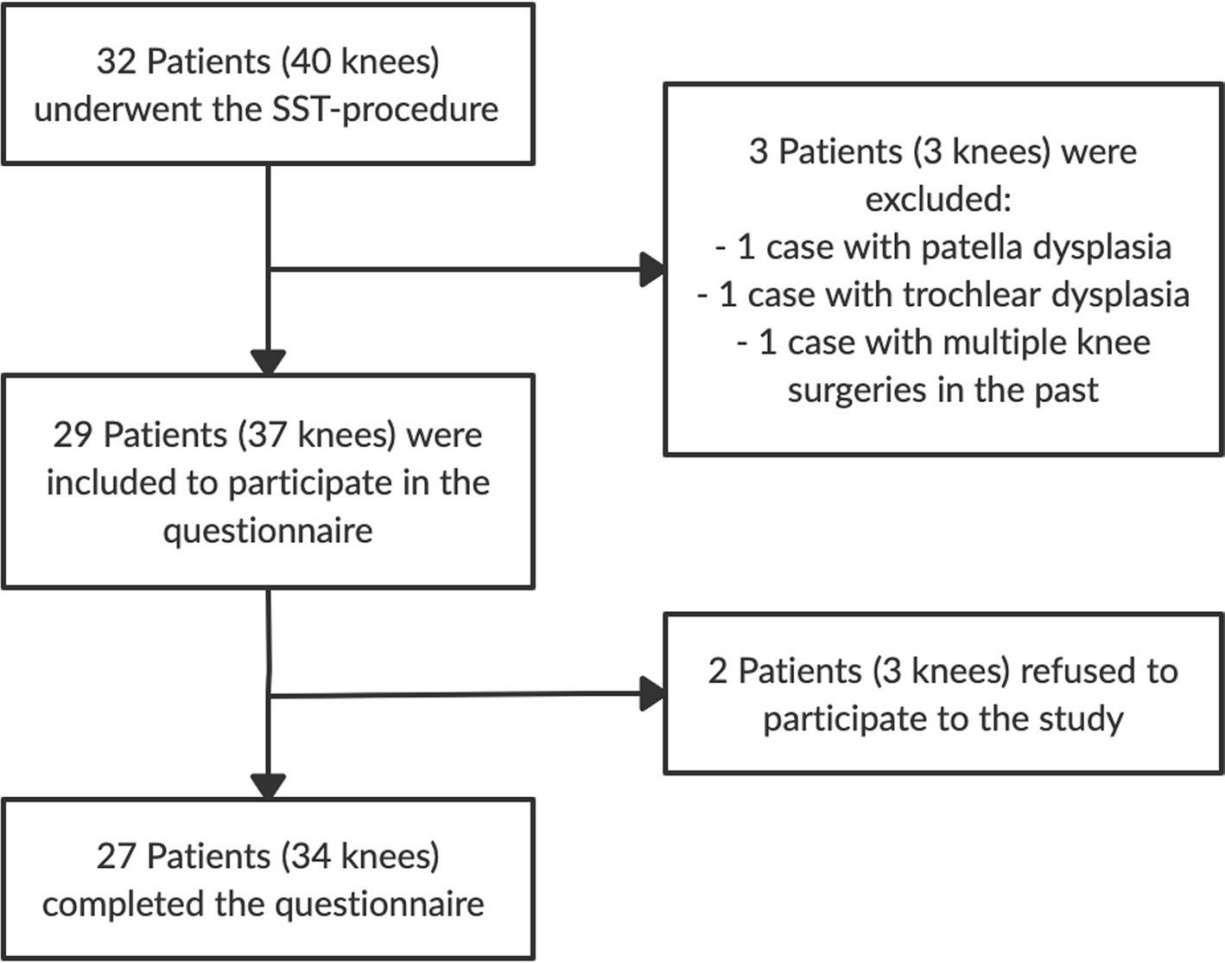
Flowchart of included patients

Between January 2001 and January 2020, a total of 32 patients were treated with the SST procedure. Eight patients were operated on both knees, resulting in a total of 40 operated cases that were considered for inclusion. Three cases were excluded based on the exclusion criteria (Fig. 2). Twenty-nine patients (37 knees) were contacted to participate in the study. Two patients did not want to participate, resulting in 27 patients (34 knees) who completed the questionnaires and were included in the final analysis. Baseline characteristics of the overall group and the groups before and after 2010 are reported in Table 1. The mean overall follow-up was 10.4 ± 4.8 years (range 3–19). The group that was operated before 2010 had a significant longer mean follow-up period compared with the group that was operated after 2010 (14.1 vs 6.6 years, *p* = 0.02).

**Table 1:** Patient demographics

	Overall	Operated before 2010	Operated after 2010	*P*-value
Patients (knees)	27 (34)	14 (17)	14 (17)	1.00
Age at operation, mean ± SD	23.7 ± 11.3	20.1 ± 8.5	27.3 ± 12.7	0.24
Follow-up in years, mean ± SD	10.4 ± 4.8	14.1 ± 3.5	6.6 ± 2.5	**0.02**
Male, No of patients (%)	3 (11%)	1 (6%)	2 (14%)	
Female, No of patients (%)	24 (89%)	13 (93%)	12 (86%)	1.00
BMI, mean ± SD	24.9 ± 4.6	26.8 ± 5.4	23.6 ± 3.6	0.40
Bilateral cases, n (%)	7 (21%)	4 (24%)	3 (18%)	0.65
Left knee, n (%)	16 (47%)	9 (53%)	7 (41%)	
Right knee, n (%)	18 (53%)	8 (47%)	10 (59%)	0.73

*n* Number of knees, *SD* Standard deviation

### Redislocations and functional outcome

Redislocations were reported in 8 cases (23.5%) and subluxations in 13 cases (38.2%) (Tables 2 and 3). Noted was that the redislocations and subluxations mainly occurred in the early years of the study. Seventeen knees were operated before January 2010 and the other 17 knees after January 2010. From the total of 8 cases that reported redislocations, 7 cases (87.5%) were operated before 2010. The redislocation rate of 5.9% in the population after 2010, was significantly smaller compared with the rate of 41.2% before 2010 (*p* = 0.04). Also, subluxations were significantly more common in the cases before 2010 (58.8%) compared with after 2010 (17.6%) (*p* = 0.03).

**Table 2:** Patient reported redislocations and subluxations

	Total of population, (*n* = 34)	Operated before 2010, (*n* = 17)	Operated after 2010, (*n* = 17)	*P*-value
Redislocations, n (%)	8 (23.5%)	7 (41.2%)	1 (5.9%)	**0.04**
Subluxations, n (%)	13 (38.2%)	10 (58.8%)	3 (17.6%)	**0.03**

*n* Number of knees

**Table 3:** Functional outcomes

	All knees	No redislocations	Redislocations		No subluxations	Subluxations	
	(*n* = 34)	(*n* = 26)	(*n* = 8)	*P*-value	(*n* = 21)	(*n* = 13)	*P*-value
IKDC subjective knee form, median (IQR)	54 (43–61)	55 (44–63)	49 (38–60)	0.34	54 (44–63)	53 (41–60)	0.57
LYSHOLM, median (IQR)	76 (56–85)	78 (63–86)	55 (46–73)	**0.046**	79 (64–87)	61 (49–81)	0.08
KUJALA, median (IQR)	81 (65–88)	83 (69–90)	69 (60–75)	0.06	84 (71–91)	73 (55–83)	0.07
TEGNER preoperative, median (IQR)	3 (2–6)	3 (2–6)	5 (1–7)	0.67	3 (2–7)	5 (2–7)	0.60
TEGNER postoperative, median (IQR)	3 (2–6)	4 (2–6)	2 (1–2)	**0.03**	1 (1–6)	2 (2–4)	0.17
SF-36							
Physical functioning, median (IQR)	78 (59–91)	85 (63–95)	75 (53–83)	0.18	85 (65–95)	70 (53–88)	0.31
Pain, median (IQR)	50 (50–70)	50 (50–75)	50 (50–69)	0.61	50 (50–88)	50 (50–63)	0.51
Physical role limitation, median (IQR)	83 (45–95)	90 (50–95)	48 (38–85)	0.052	90 (63–95)	50 (45–95)	0.15
General health perception, median (IQR)	73 (53–84)	75 (62–84)	68 (46–83)	0.50	80 (65–85)	70 (40–75)	0.14
NRS rest, median (IQR)	1 (0–4)	0 (0–4)	2 (0–6)	0.25	0 (0–3)	2 (0–5)	0.23
NRS walking, median (IQR)	3 (0–5)	3 (0–5)	4 (2–7)	0.22	3 (0–5)	5 (0–7)	0.16
NRS sports, median (IQR)	5 (3–8)	5 (3–7)	8 (5–9)	0.09	4 (2–7)	7 (4–9)	0.07
Subjective occasional swelling, n (%)				0.34			0.27
Never	16 (47%)	14 (54%)	2 (25%)		11 (52%)	5 (39%)	
Occasionally	10 (29%)	7 (27%)	3 (38%)		7 (33%)	3 (23%)	
Invalidating	8 (24%)	5 (19%)	3 (38%)		3 (14%)	5 (39%)	
Subjective instability, n (%)				**0.03**			**0.01**
Never	17 (50%)	15 (58%)	2 (25%)		14 (67%)	3 (23%)	
Occasionally	8 (24%)	7 (27%)	1 (13%)		5 (24%)	3 (23%)	
Invalidating	9 (26%)	4 (15%)	5 (63%)		2 (10%)	7 (54%)	
Subjective crepitus, n (%)				0.26			0.65
Never	11 (32%)	9 (35%)	2 (25%)		8 (38%)	3 (23%)	
Occasionally	5 (15%)	5 (19%)	0 (0%)		3 (14%)	2 (15%)	
Invalidating	18 (53%)	12 (46%)	6 (75%)		10 (48%)	8 (62%)	

*IQR* Interquartile range, *n* Number of knees

The functional outcome scores are presented in Table 3. Focusing on the overall clinical outcome, the IKDC score was 54. The Lysholm and Kujala questionnaires scored 76 and 81. The preoperative Tegner activity level was 3.0, where no improvement in score was seen at follow-up. Daily pain at follow-up was assessed by means of the SF 36 questionnaire and NRS score. A median NRS score of 1 was found in rest, but this score increased for walking and during sports (score of 3 and 5). Subjective scores for incidental oedema, instability and crepitus were found as at least “occasionally” in 50% of all cases. The subjective satisfactory scores are presented in Table 4. When looking at these scores, 2 cases (5.9%) rated their satisfactory as ‘bad’ after surgery. Daily functioning after surgery was worsened in 4 cases (11.7%). A greater pain intensity at follow-up was felt in 6 cases (17.6%).

**Table 4:** Subjective satisfactory

	All knees	With redislocation	No redislocation	*P*-value	With subluxation	No subluxation	*P*-value
Satisfactory after surgery							
Excellent, n (%)	11 (32.4%)	2 (25.0%)	9 (34.6%)		2 (15.4%)	9 (42.9%)	
Good, n (%)	16 (47.1%)	3 (37.5%)	13 (50.0%)		5 (38.5%)	11 (52.4%)	
Reasonable, n (%)	5 (14.7%)	1 (12.5%)	4 (15.4%)		5 (38.5%)	0 (0.0%)	
Bad, n (%)	2 (5.9%)	2 (25.0%)	0 (0.0%)	0.27	1 (7.7%)	1 (4.8%)	**0.02**
Change in daily functioning							
Much improved, n (%)	6 (17.6%)	1 (12.5%)	5 (19.2%)		2 (15.4%)	4 (19.0%)	
Improved, n (%)	7 (20.6%)	0 (0.0%)	7 (26.9%)		1 (7.7%)	6 (28.6%)	
Slightly improved, n (%)	7 (20.6%)	3 (37.5%)	4 (15.4%)		4 (30.8%)	3 (14.3%)	
Not changed, n (%)	10 (29.4%)	1 (12.5%)	9 (34.6%)		4 (30.8%)	6 (28.6%)	
Slightly worse, n (%)	3 (8.8%)	2 (25.0%)	1 (3.8%)		1 (7.7%)	2 (9.5%)	
Much worse, n (%)	1 (2.9%)	1 (12.5%)	0 (0.0%)	0.13	1 (7.7%)	0 (0.0%)	0.38
Change in pain intensity							
Much improved, n (%)	7 (20.6%)	1 (12.5%)	6 (23.1%)		2 (15.4%)	5 (23.8%)	
Improved, n (%)	11 (32.4%)	0 (0.0%)	11 (42.3%)		3 (23.1%)	8 (38.1%)	
Slightly improved, n (%)	5 (14.7%)	3 (37.5%)	2 (7.7%)		2 (15.4%)	3 (14.3%)	
Not changed, n (%)	5 (14.7%)	0 (0.0%)	5 (19.2%)		2 (15.4%)	3 (14.3%)	
Slightly worse, n (%)	5 (14.7%)	3 (37.5%)	2 (7.7%)		3 (23.1%)	2 (9.5%)	
Much worse, n (%)	1 (2.9%)	1 (12.5%)	0 (0.0%)	**0.03**	1 (15.4%)	0 (0.0%)	0.16

*n* Number of knees

The occurrence of redislocations resulted in a lower functional outcome score with higher pain levels. No significant differences were found between the RD and NRD group concerning age at time of surgery, BMI and gender. The NRD group scored better on all questionnaires with significantly higher marks on the Lysholm (*p* = 0.046) and postoperative Tegner score (*p* = 0.03). The subjective score for instability was also significantly less in the NRD group versus the RD group (42% versus 76%, *p* = 0.03).

When comparing the subluxation group with the no-subluxation group no significant differences were observed with regard to functional outcome and pain. The subjective occurrence of instability was the only variable that was significantly higher in the subluxation group (77% vs 34%, *p* = 0.004).

The subjective satisfaction towards the surgery was significantly better in the no subluxation group compared to the subluxation group (*p* = 0.02) (Table 4). Also, the NRD group experienced significantly less pain than the RD group at follow-up (*p* = 0.03). The satisfactory scores between the RD and NRD group and the scores for change in daily functioning between the RD, NRD, subluxation and no subluxation groups were not significant. Two cases (25%) in the RD group and 1 case (7.7%) in the subluxation group were not satisfied with the surgery at follow-up. Additionally, 50% of the RD group and subluxation group did experience improvement in their daily functioning.

### Complications and revisions

Major complications such as infection, deep venous thrombosis or embolism were not observed during the study period. In 3 patients a secondary surgical procedure was needed. One patient who had been operated on before 2010 underwent a successful tuberosity transposition, because of patellar redislocations. The other 2 patients were operated on after 2010 and received a revision of the SST-procedure. One patient had a detachment of the distal lateral PT transposition. In the other patient, the strip of the medial retinaculum became insufficient due to 2 other knee surgeries. No revisions were performed in other hospitals.

## Discussion

The most important finding of this study is the significant difference in redislocations and subluxations rates between the populations before and after 2010. The SST-procedure after 2010 serves it purpose of stabilizing the patella in patients with recurrent instability of the patella. This study aimed to report and evaluate an alternative stabilizing soft-tissue surgical technique, the SST-procedure, for patients with recurrent patellar dislocations. To our knowledge, this is the first report that describes the SST-technique as a four-in-one technique and evaluates the outcome.

In the literature, several of soft tissue techniques are described to treat patella instability. Other widely used procedures are the 3-in-1 technique and the Roux-Goldthwait procedure. The SST-procedure differs from the 3-in-1 technique [13, 15] by reinforcing the MPFL by a medial retinaculum strip instead of advancing the vastus medialis muscle. Also, after 2010 the lateral quarter of the PT is used, whereas the 3-in-1 technique uses the medial half of the PT. The choice in 2010 to use the lateral quarter instead of the lateral third of the PT was theoretically based on the assumption that this would cause less lateral patellar tilt. The Roux-Goldthwait [12, 18] technique on the other hand uses the lateral half of the PT. The SST-procedure combines these techniques with an MPFL reinforcement by using a strip of the medial retinaculum and by transferring the distal lateral quarter of the PT to the medial side. The combination of these techniques and without the use of a semitendinosus/gracilis or allograft, is intended to offer more patellofemoral stability in the least invasive way, without causing too much lateral patellar tilt and posterior patellofemoral pressure.

After a mean follow-up of 10.4 ± 4.8 years (range 3–19), an overall redislocation rate of 23.5% and subluxation rate of 38.2% was observed after the SST-procedure. However, the incidence decreased significantly after 2010 (5.9%), after the slight change of the surgical technique. Previous studies show a wide range of redislocation from 0 – 14% (Table 5) [10, 12, 13, 15, 18, 23, 24]. Only Ma et al. [11] found a redislocation rate of 31% after medial capsule reefing and a rate of 6.2% after a medial patellar retinaculum plasty. It was noticeable that all functional and subjective outcome scores were significantly higher in the medial patellar retinaculum plasty group. A recent systematic review of Longo et al. [9] that compared different procedures (including tibial tubercle osteotomy, Elmslie-Trillat, Roux Goldthwait, Fulkerson and Maquet-procedures) for managing patellar dislocations, found an overall redislocation rate of 7%. This is consistent with the systematic review of Baumann et al. [2] who found a redislocation rate of 2–10%. They only included studies that reconstructed the medial patellotibial ligament (MPTL) and medial patellofemoral ligament (MPFL). MPFL reconstruction techniques have a high rate of success for patients with patellofemoral instability, however, also high complication rates (26.1%) are known with this procedure [17]. Moreover, several kinds of tunnelled MPFL reconstructions are contraindicated in paediatric populations due to their growth plates [11]. In contrast, the SST procedure is a proximal and distal realignment procedure using soft tissues only, which makes the procedure possibly also more suitable for patellar dislocations in paediatric populations. However, based on the patients operated before and after 2010, we think that transferring the distal lateral third of the PT is possibly associated with the significant higher redislocation rate. We speculate that using a third of the lateral PT will cause more medializing force, however, it may also result in a lateral patellar tilt, which can cause redislocations. The medializing force that is reduced by using a of the lateral PT instead of a third, is likely to be compensated by the other medializing techniques that are part of the SST-procedure. Future comparative or biomechanical studies are needed to compare the degree of lateral patellar tilt, patellar traction and difference in redislocations after using a third or a quarter of the PT in the SST-procedure. Another reason for the discrepancy between the two groups, may be the significantly longer follow-up period of the group before 2010.

**Table 5:** Characteristics and outcomes of comparable studies

Author	Study design	No of Knees	Procedure	Population	Follow-up in years	Outcomes
Myers 1999 [13]	Cohort, retrospective	42	3-in-1 procedure ^a^	21.1 ± 9.9 Years, RPD	3.7 ± 1.2	Redislocations: 9.5%
Sillanpaa 2008 [18]	Cohort, prospective	A. 18 B. 29	A. MPFL-reconstruction with adductor magnus tenodesis B. Roux Goldthwait ^b^	20.0 ± 1.1 Years, RPD	10 ± 1.3	Redislocations: A: 7% and B: 14% Kujala: A: 88 and B: 86 Postoperative Tegner: A: 4 and B: 5
Oliva 2009 [15]	Cohort, prospective	25	3-in-1 procedure ^a^	13.5 ± 3.8 Years, RPD	3.8 ± 0.9	redislocations: 0% Kujala: 94
Luhmann 2011[10]	Cohort, prospective	27	Medial retinacular reefing	14.1 ± 2.3 Years, RPD or subluxations	5.1 ± 1.1	Redislocations: 7.4% IKDC: 65, Lysholm: 69 Postoperative Tegner: 5.4
Ma 2012 [11]	RCT	A. 29 B. 32	A. Medial capsule reefing B. Medial patellar retinaculum plasty	13.5 ± 1.5 Years, RPD with MPFL injury	4.2 ± 0.9	Redislocations: A: 31% and B: 6.2% Kujala: A: 78 and B: 82
Zhao 2012 [24]	RCT	A. 43 B. 45	A. Medial retinaculum plication B. MPFL-reconstruction with semitendinosus tendon	25.5 ± 5.5 Years, RPD or instability	5.0	Redislocations: A: 9.3% and B: 2.2% IKDC: A: 61 and B: 79, Lysholm: A: 69 and B: 87, Kujala: A: 74 and B: 87 Preoperative Tegner: A: 2.9 and B: 3.1 Postoperative Tegner: A: 4 and B: 5.7
Malecki 2016 [12]	Cohort, prospective	A. 32 B. 33	A. MPFL-reconstruction with adductor magnus tendon B. Roux Goldthwait ^b^ with vastus medialis advancement	14 ± 2.5 Years, RPD	5.6 ± 2.5	Lysholm: A: 90, B: 84 Kujala: A: 91, B: 85
Yang 2019 [23]	Cohort, prospective	58	Reconstruction of MPFL and MTFL with semitendinosus tendon	22.6 ± 4.9 Years, RPD with patella alta	3.0 ± 0.9	IKDC: 85 Kujala: 90

*RPD* Recurrent patellar dislocations, *MPFL* Medial patellofemoral ligament, *MPTL* Medial patellotibial ligament

^a^ 3-in-1 procedure: 1) Lateral retinaculum release 2) vastus medialis advancement 3) transfer of the distal medial third of the PT to the medial collateral ligament and tibia

^b^ Roux Goldthwait: 1) Lateral retinaculum release 2) release of the distal lateral half of the PT 3) transfer behind the medial half of the PT and attached to the tibia

The functional outcome scores of the Kujala and Lysholm are in accordance with previous comparable studies as can be seen in Table 5 [10–13, 15, 18, 23, 24]. The IKDC and postoperative Tegner score in these studies range from 61 to 85 and 4 to 5.7 and are slightly superior compared with our results. Possibly, these differences are found due to heterogeneity between the studies, whereas they differ in techniques, patient population and length of follow-up. The lack of functional improvement between our pre and postoperative Tegner scores may be due to the higher pain scores found in our population. The NRS score in this study is 1 at rest but increases while walking and doing sports to 3 and 5. This increase of pain probably influences patients their daily activities, which results in a lower postoperative Tegner score in comparison with other studies [10, 18, 24].

Ongoing pain complaints after patella realignment procedures are commonly described in the literature [5, 10, 18]. Sometimes, a too tight construction of the ligaments is mentioned as the reason of the pain, but this is expected to diminish due to the ligaments that become slowly laxer after a longer period of time [12, 14]. Another reason could be the development of a lateral patellar tilt, which may cause osteoarthritis [9]. However, it was therefore decided in 2010 to use a quarter of the PT instead of a third. Finally, the ongoing pain could be a result of the initial surgical indication. Because the study is retrospective and there is no data of a radiographic assessment of lower leg alignment. Future work should consider to prospectively investigate the potential outcome of the SST-procedure after an extensive assessment of the upper and lower leg alignment to determine the best surgical indication.

### Limitations

There are several limitations in this study that must be acknowledged. First, interim follow-up moments with radiographic assessments to explain what caused the higher redislocation and subluxation rates in the early years of the study are missing. However, the significant difference that has been found, strongly suggests that the change in the surgical protocol caused the decreased rates. Secondly, the study design was retrospective, therefore, we were not able to report the exact preoperative classifications of the skeletal deformities of patients that were included for the SST-procedure. Thirdly, preoperative data from the IKDC, Kujala and Lysholm were unknown. Nevertheless, several questions were asked about pain intensity and daily functioning since the operation, in order to give a good impression of whether the operation has caused improvement. Given the fact that many techniques are described and analysed in the literature, without consensus about the best surgical treatment of patients with RPD, it remains a challenging task to provide evidence for the most efficient way of treating patients with RPD.

## Conclusion

In conclusion, the SST-procedure for patients with recurrent patella dislocations seems to be a safe procedure, with a similar redislocation rate based on the cases after 2010. Since no osseous corrections are performed in the SST procedure, the procedure appears to be suitable for the paediatric and adolescent population.

## Abbreviations

RPD: Recurrent patellar dislocations
SST-procedure: Spaarne Soft Tissue procedure
MPFL: Medial Patellofemoral ligament
MPTL: Medial Patellotibial ligament
PT: Patellar tendon
RD: Redislocation
NRD: No-redislocation
